# Investigation of the Mechanical Properties of the Human Osteosarcoma Cell at Different Cell Cycle Stages †

**DOI:** 10.3390/mi8030089

**Published:** 2017-03-15

**Authors:** Guocheng Zhang, Na Fan, Xiaoying Lv, Yiyao Liu, Jian Guo, Longxiang Yang, Bei Peng, Hai Jiang

**Affiliations:** 1 School of Mechatronics Engineering, University of Electronic Science and Technology of China, Chengdu 611731, Sichuan, China; 201511080106@std.uestc.edu.cn (G.Z.); na_fan@uestc.edu.cn (N.F.); jguo@xtu.edu.cn (J.G.); 201411080108@std.uestc.edu.cn (L.Y.); 2 Center for Robotics, University of Electronic Science and Technology of China, Chengdu 611731, Sichuan, China; 3 School of Life Science and Technology, University of Electronic Science and Technology of China, Chengdu 611731, Sichuan, China; 201521090306@std.uestc.edu.cn (X.L.); liuyiyao@uestc.edu.cn (Y.L.); 4 School of Materials Science and Engineering, Xiangtan University, Xiangtan 411105, Hunan, China

**Keywords:** mechanical property, elastic modulus, atomic force microscopy, human osteosarcoma cells, cell cycle

## Abstract

The mechanical properties of a single cell play substantial roles in cell mitosis, differentiation, and carcinogenesis. According to the difference of elastic modulus between the benign cell and the tumor cell, it has been shown that the mechanical properties of cells, as special biomarkers, may contribute greatly to disease diagnosis and drug screening. However, the mechanical properties of cells at different cell cycle stages are still not clear, which may mislead us when we use them as biomarkers. In this paper, the target regions of the human osteosarcoma cell were precisely scanned without causing any cell damage by using an atomic force microscopy (AFM) for the first time. Then, the elasticity properties of the human osteosarcoma cells were investigated quantitatively at various regions and cell cycle stages. The 32 × 32 resolution map of the elasticity showed that the elastic modulus of the cells at the interphase was larger than that at the telophase of mitosis. Moreover, the elastic modulus of the cell in the peripheral region was larger than that in the nuclear region of the cell. This work provides an accurate approach to measure the elasticity properties of cells at different stages of the cell cycle for further application in the disease diagnosis.

## 1. Introduction

The mechanical properties of the cell change with the alteration of the cell function; these properties play important roles in adhesion, migration, mitosis and differentiation [1,2]. For instance, the tumorigenesis and oncogenic progression may lead to mechanical and structural variations. Therefore, the mechanical properties of cells have been employed as the biomarkers in the cancer diagnosis [3,4].

Numerous methods have been developed to study the mechanical properties of cells, including micropipette aspiration [5], utilization of optical tweezers [6], magnetometric analysis [7] and atomic force microscopy (AFM)-based methods [8]. Jiang et al. [9] measured the regional cell stiffness with optical magnetic twisting cytometry and investigated the relationship between the cytoskeleton and the mechanical properties of the primary human aortic smooth muscle cells. Rebelo et al. [10] studied the viscoelastic properties of cells from different kidney cancer phenotypes by using AFM and conducted the elastic modulus map. They analyzed the elastic modulus distribution of cells and found that the non-tumorigenic cells were less deformable but more viscous than the cancerous cells. Cross et al. [11] investigated the nanomechanical responses of metastatic cancer cells and benign mesothelial cells by using AFM and the results showed that the metastatic tumor cells were more than 80% softer than the benign cells. Costa et al. [12] reported that the periphery of bovine pulmonary artery endothelial cells was two or three times as stiff as that of the cell body.

However, variation in the mechanical properties of cells could also occur at the different cell cycle stages, which might mislead us when we use them as biomarkers. The mechanical properties of the eukaryotic cells are dominated by their cytoskeleton, which may experience huge changes and remodelling during the cell cycle [13,14,15]. Usually, the actin filaments dominate the mechanic properties of the cell. At the interphase, the cell includes an extensive actin network. However, once the cell enters the mitosis stage, the network will be deconstructed and rearranged. For most cells, the shape of the cell may undergo a process from spindle to round with furrows during the cell cycle until the cell divides into two daughter cells [16,17,18]. The aforementioned studies neglected the variations of the state and shape of the cell during the cell cycle which has an obvious effect on the mechanical properties of cells. Therefore, the investigation of accurate and practical measurement of mechanical properties at the different cell cycle stages is immensely significant. For example, Stewart et al. [19] introduced an approach to measure the mechanical properties of globular cells during the mitosis by using the AFM. They found that the mechanical stress at the interphase was higher than that at the anaphase.

In this paper, the elasticity properties of the human osteosarcoma (U-2 OS) cells at the different cell cycle stages and the relationship between the elastic modulus and the indentation depth were investigated quantitatively by AFM in the force–volume mode. In addition, the elastic modulus of the nuclear and peripheral regions of the U-2 OS cells were surveyed at the interphase and the telophase of mitosis. To our knowledge, this is the first time that the elastic properties at the different regions of the cell and the different cell cycle stages have been compared. This work also provides an accurate approach to measure the elasticity properties of cells at the different cell cycle stages. Such findings can be applied in the disease diagnosis and provide a strong reference.

## 2. Methods and Materials

### 2.1. Cell Culture

U-2 OS cells (ATCC^®^) were cultured in McCoy’s 5A Medium and supplemented with 10% fetal bovine serum (*v*/*v*, Gibco, Thermo Fisher Scientific, Waltham, MA, USA) at 37 °C under 5% CO_2_ atmosphere. The cells were seeded onto poly-l-lysine-coated 35-mm Petri dishes. The test was conducted when the cell density increased to approximately 30%. Before testing, the cells were washed with phosphate-buffered saline (PBS) three times to remove the unattached cells and organic medium. They were finally re-suspended in 1 mL PBS buffer for living cell imaging and elasticity mapping experiments.

### 2.2. Actin and Nucleic Acid Staining

Actin and nucleic acid staining were used for identifying the morphology of the cell nucleus and F-actin, and the shapes at different cell cycle stages. DNA and actin were stained with 4’,6-diamidino-2-phenylindole dihydrochloride (DAPI) and phalloidin (Sigma-Aldrich, St. Louis, MO, USA), respectively. Cells were fixed in 4% paraformaldehyde (Sigma-Aldrich) for 15 min at 4 °C and permeabilized with 0.1% Triton X-100, 1% Bovine Serum Albumin (BSA) (Sigma-Aldrich). Next, cells were incubated with Phalloidin and DAPI for 1 h at 4 °C in the dark. Confocal images were collected on an Olympus Fluoview FV1000 microscope (Olympus, Tokyo, Japan).

### 2.3. Cell Imaging by Atomic Force Microscopy (AFM)

The morphologies of cells were scanned by an AFM (Agilent 5500, Agilent Technologies, Santa Clara, CA, USA) with a maximum *x-y* scan range of 90 μm and a *z* range of 7 μm in contact mode. The curvature radius of the silicon nitride tips (MSCT–AUHW, Veeco, Town of Oyster Bay, NY, USA) *R* is approximately 10 nm and the spring constant *k* of the rectangle cantilever is calibrated to be ~0.02 N/m by the thermal noise method [20]. Before the experiments, the silicon nitride tips were exposed to ultraviolet light for 0.5 h to eliminate the organic contaminates so as to reduce the measurement error. The topographic images and deflection images of cells were recorded during the tips canning process, respectively.

### 2.4. AFM Force Spectroscopy Analysis

From the force spectroscopy obtained by AFM, the elastic modulus of cells can be obtained by the Hertz–Sneddon model [21]. The elastic modulus of the cell *E* was calculated by the following equation because the pyramidal Si_3_N_4_ tip was used in the experiment:

(1) $$F=\frac{2}{\pi }\frac{E}{1-\nu^{2}}\tan\partial \delta^{2}$$

where *F* is the normal force, *v* is Poisson’s ratio (about 0.5 used in cell [22]), *ә* is the half-open angle of the pyramidal Si_3_N_4_ tip, and δ is the probe indentation depth of the tip. The indentation depth δ can be obtained from δ = Δ*z* − Δ*d* (Δ*z* = *z* − *z*_0_ is the piezo-actuator displacement and Δ*d* = *d* − *d*_0_ is the cantilever deflection, where *d* and *z* represent the cantilever deflection and piezo-displacement respectively). (*z*_0_, *d*_0_) represents the contact point. In this study, the elasticity mapping experiments were conducted by using 10 individual cells and five pairs of daughter cells in the force–volume mode with a resolution of 32 × 32 (32 × 32 measuring points for force–distance curves). This specification allowed us to obtain simultaneously the force–distance curves in every area of the single cell. All measurements were taken at a scan rate of 1 Hz.

## 3. Results and Discussion

### 3.1. Analysis of Elastic Modulus under Different Indentation Depths

**Figure 1:**
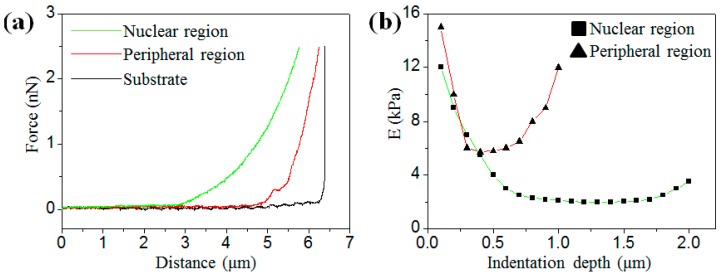
(**a**) Three representative force–distance curves obtained from the substrate, the nuclear and peripheral regions of an osteosarcoma cell, respectively; (**b**) the relationship between the elastic modulus and the indentation depth at the nuclear and peripheral regions, respectively.

To obtain the accurate results of the elastic modulus in the different regions of cells, the force (indentation depth) must be appropriate, otherwise, the AFM probe will have a big chance to contact the nucleus or the substrate, resulting in measurement errors. Figure 1a shows three representative force–distance curves obtained from the substrate, the nuclear and the peripheral regions of an osteosarcoma cell, respectively. In this study, the peripheral and nuclear regions are defined by the height (*H*) of the cell area (peripheral region: *H* < 1 μm; nuclear region: *H* > 1 μm). To ensure the accuracy of measurement, the force–distance curves were fitted under a series of indentation depths by the Hertz–Sneddon model. Figure 1b demonstrates the relationship between the elastic modulus and the indentation depth which is extracted from the force–indentation curves shown in Figure 1a. As reported previously, the variation of the elastic modulus with indentation depth is mainly ascribed to the distribution of the cytoskeleton of the cell [23,24]. In the nuclear region (red line) as shown in Figure 1b, the elastic modulus *E* starts with 12 kPa at the indentation depth of 0.1 μm, then drops quickly to less than 4 kPa at the indentation depth of 0.5 μm. After that, the elastic modulus remains at the level of ~1.9 kPa over the range from 0.8 μm to 1.7 μm. The elastic modulus increases slightly when the indentation depth was larger than 1.7 μm because the AFM and the nucleus are in contact. Conversely, in the the peripheral region (green line) as shown in Figure 1b, the elastic modulus remained at the level of ~6 kPa over a small range around 0.5 μm, and then increases dramatically. The reason for this is the contact between the AFM probe and the substrate. Therefore, in the following tests, the loading force was set up at 0.5 nN (the indentation depth was around 500 nm) to eliminate the effect of the substrate or the nucleus.

### 3.2. AFM Images of Cells at the Different Cell Cycle Stages

**Figure 2:**
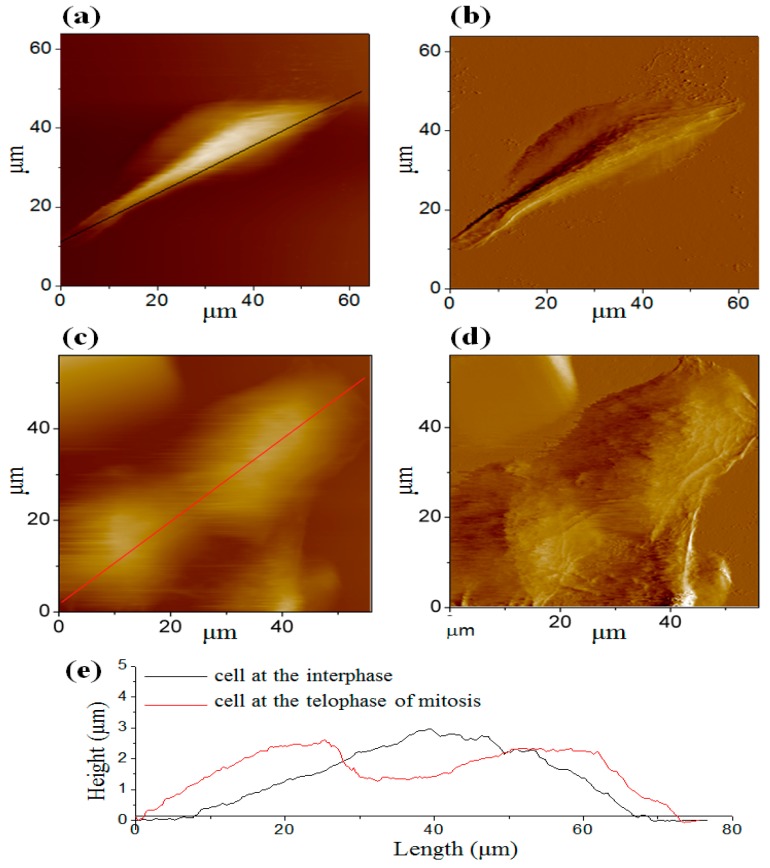
Atomic force microscopy (AFM) images of the osteosarcoma cell. (**a**) Topographic image of the cell at the interphase; (**b**) deflection image of the cell at the interphase; (**c**) topographic image of the cell at the telophase of mitosis; (**d**) deflection image of the cell at the telophase of mitosis; (**e**) two cross-sectional profiles of the cell shown in (**a**) and (**c**) (the positions of the cross-sections were marked by black and red lines, respectively).

**Figure 3:**
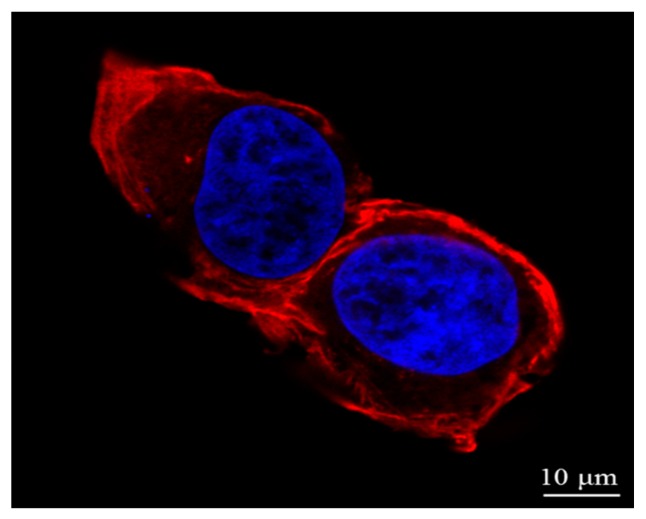
Immunofluorescence image of two daughter cells at the telophase of mitosis. The F-actin and the nucleus were stained with Phalloidin (red) and 4’,6-diamidino-2-phenylindole dihydrochloride (DAPI) (blue), respectively.

Figure 2a,b shows a single osteosarcoma cell at the interphase when plenty of DNA and proteins are synthesized and the cell spreads completely. Typically, the interphase lasts for at least 90% of the entire time of the cell cycle. Once the flat lamellipodia is observed at the peripheral region, the cell adheres tightly on the substrate. Figure 2c,d shows the cell at the telophase of mitosis after a mother cell has divided into two daughter cells. No flat lamellipodia is observed at the peripheral region. Two cross-sectional height profiles (Figure 2e) are obtained from the positions of the marked lines in Figure 2a,c. As shown in Figure 2e, the black curve indicates that the nucleus is in the center of the cell during the interphase. When it proceeds to the telophase of mitosis, a cleavage furrow (red curve) which is an important sign of the final stage of mitosis is clearly visible [25]. The cell membrane in the vicinity of the equatorial plate concaves inward, and the cell is split into two daughter cells. To prove this, another osteosarcoma cell was stained at the telophase of mitosis as shown in Figure 3. It is obvious that the immunofluorescence image highly corresponded to the AFM image.

### 3.3. Elastic Modulus Map of the Cell at the Interphase

**Figure 4:**
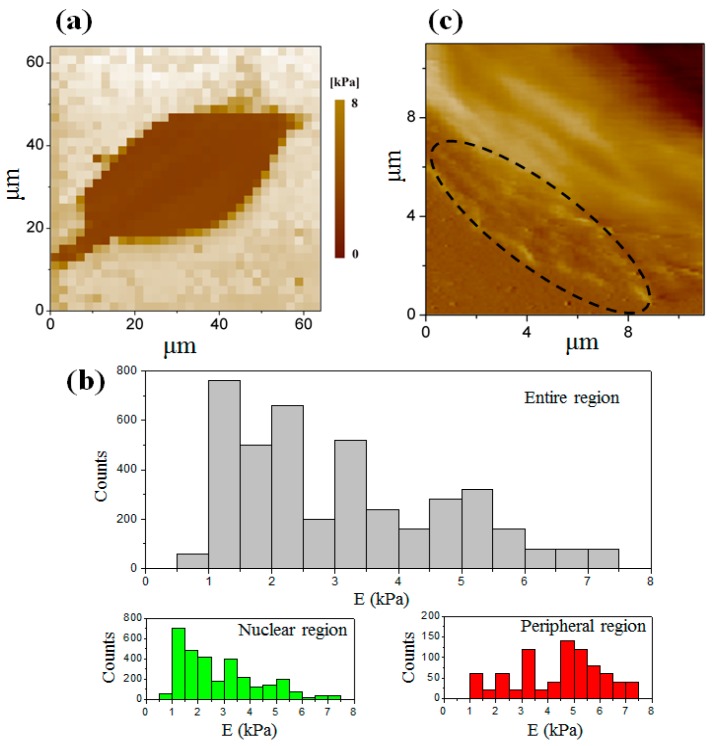
(**a**) The elastic modulus map of a single cell at the interphase with the resolution of 32 × 32 data points; (**b**) the histogram of the elastic modulus distribution in the whole region (gray), in the nuclear region (green), and in the peripheral region (red); (**c**) large lamellipodia area surrounded by black dashed ellipse in the peripheral region.

Figure 4a shows an elastic modulus map with 32 × 32 measuring points of a single osteosarcoma cell at the interphase (Figure 2a). It reveals the distinction of the elastic modulus in both the entire region of the cell (including the nuclear region and peripheral region) and the substrate by the color change. The nuclear region was softer than the peripheral region. Figure 4b shows the histogram of the detailed elastic modulus distribution obtained by calculating 4100 force–distance curves chosen from the elastic modulus maps of 10 individual cells. The elastic modulus of the cell in the nuclear region at the interphase ranged from 0.50 kPa to 7.5 kPa with an average value of 2.8 ± 1.5 kPa. Its elastic modulus was less than that in the peripheral region, which ranged from 1.0 kPa to 7.5 kPa with an average value of 4.4 ± 1.6 kPa.

**Figure 5:**
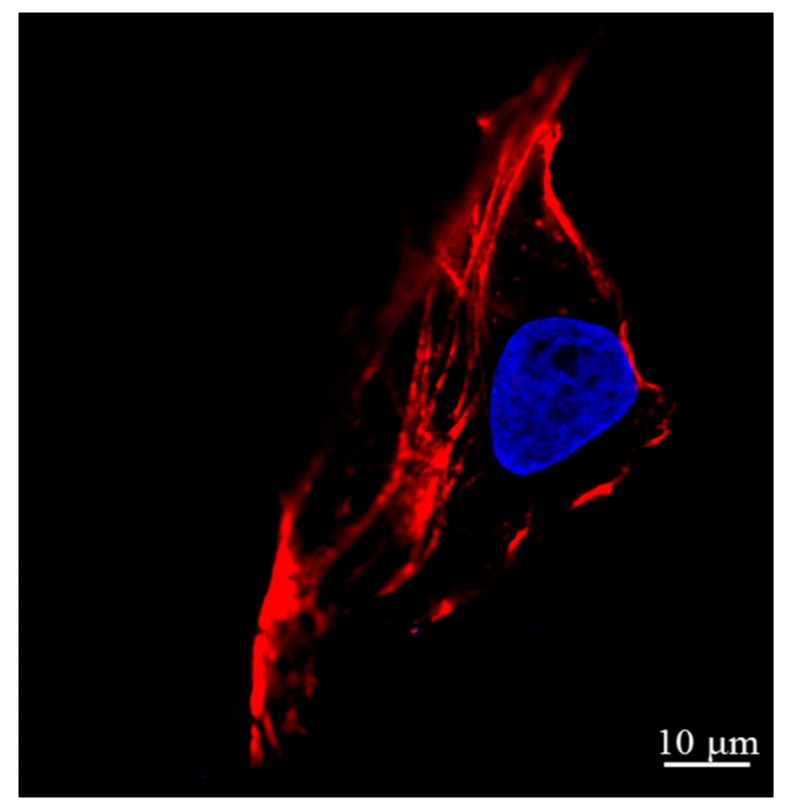
Immunofluorescence image of a single cell at the interphase. The F-actin and the nucleus were stained with Phalloidin (red) and DAPI (blue), respectively.

As mentioned above, the mechanical properties of the cell depend on the actin filaments of the cytoskeleton. The actin filaments include long stress fibers and short actin filaments, some of which are located beneath the cell membrane and the others may extend all over the entire cell body [26,27]. In this experiment, the indentation depth was much greater than the thickness of the cell membrane. Therefore, it explained that the difference of elastic modulus in distinct regions of the cell was generated due to the distribution of actin filaments in the cell body. To validate this, the actin filaments and the nucleic acid of an osteosarcoma cell were stained with phalloidin and DAPI, respectively. The red color demonstrates the distribution of the actin filaments in the cell, as shown in Figure 5. It is found that the actin filaments are highly concentrated in the peripheral region. In addition, large lamellipodia areas in the peripheral region are observed in Figure 4c, which helps to promote cell spreading and drive retrograde flow. The formation of pseudopodia also resulted from the recombination of the skeleton, especially the actin filaments which were stiffer than the intermediate filaments and microtubules appearing in the cell body [28,29].

### 3.4. Elastic Modulus Map of the Cell at the Telophase of Mitosis

**Figure 6:**
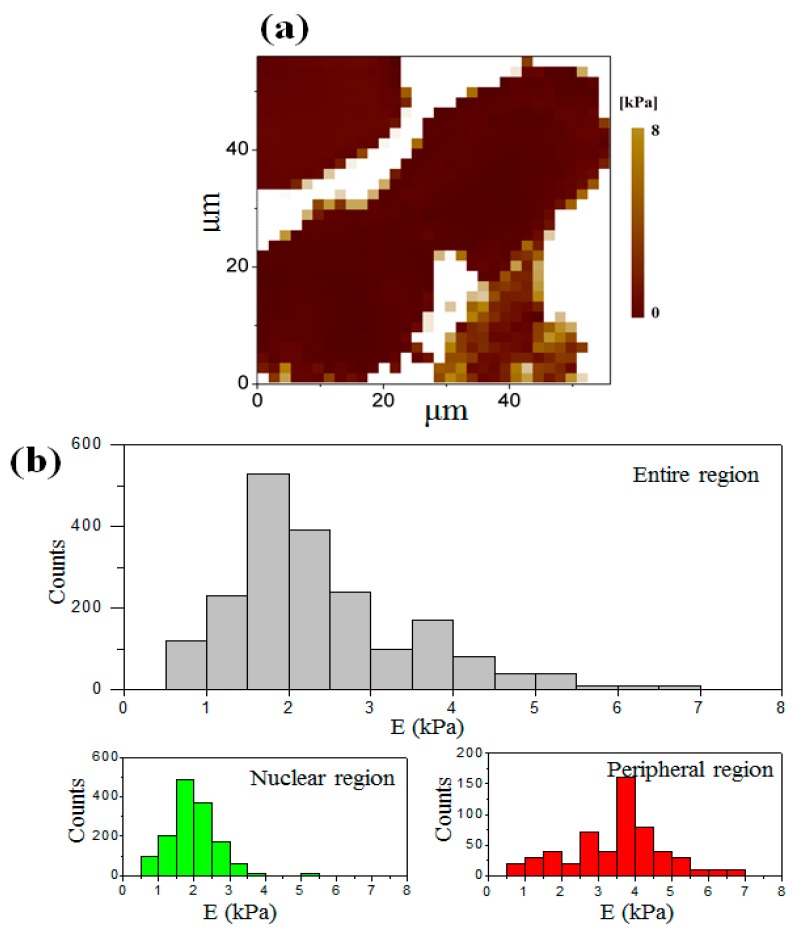
(**a**) The elastic modulus map of a single cell at the telophase of mitosis with a resolution of 32 × 32; (**b**) the histogram of the elastic modulus distribution of one daughter cell in the whole region (gray), the nuclear region (green), and the peripheral region (red).

Figure 6a illustrates the elastic modulus map of two daughter cells at the telophase of mitosis (see Figure 2c). No significant difference of the elastic modulus is measured between the two daughter cells in both the nuclear region and the peripheral region. Figure 6b is the histogram of the elastic modulus distribution obtained by calculating 1970 force–distance curves chosen from five elastic modulus maps (five pairs of daughter cells), respectively. The elastic modulus of the daughter cell in the nuclear region at the telophase of mitosis ranges from 0.5 kPa to 5.5 kPa, with an average value of 2.0 ± 0.68 kPa. Consistent with the cells at the interphase, the elastic modulus is stiffer in the peripheral region varying from 0.5 kPa to 7.0 kPa with an average value of 3.5 ± 1.3 kPa. In addition, the cleavage furrow is found to contribute significantly to the stiffness due to the high concentration of the F-actin in the furrow region [9,30].

### 3.5. Comparison of the Elastic Modulus between Two Stages

**Figure 7:**
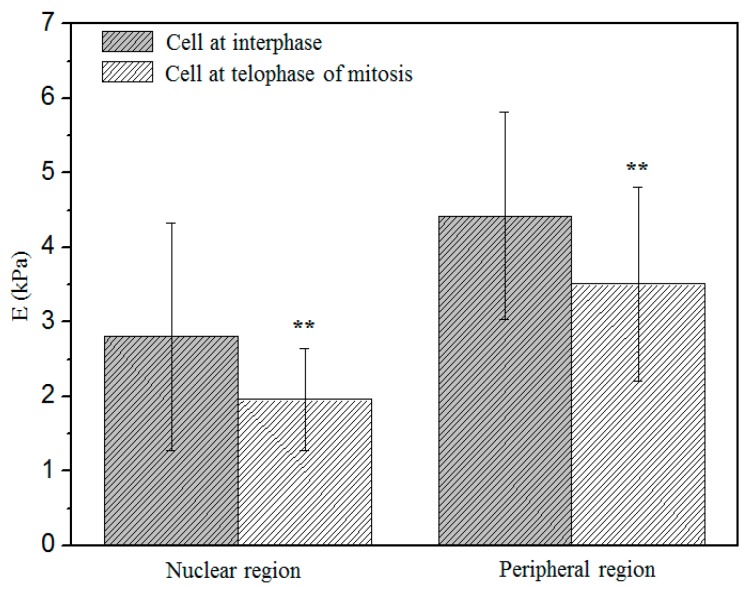
Comparison of the elastic modulus of cells at the interphase and the telophase of mitosis. Data are presented as mean ± standard error of the mean (** denoted *p* < 0.01).

A comparison of the average elastic modulus between the two stages is shown in Figure 7. A significant distinction of the average elastic modulus was obtained between the two stages in the nuclear and peripheral regions, respectively. The average elastic modulus at the interphase was higher than that at the telophase of mitosis in both the nuclear region and the peripheral region. The major reason is that the cell has an unstable structure at the telophase of mitosis due to the decreasing of the adhesion between the cell and the extracellular matrix [16], which cannot bear high amounts of stress. Moreover, in this experiment, the spreading area of the cell at the interphase (Figure 2a) was measured to be about 1500 μm^2^, whereas the spreading area of the single daughter cell at the telophase of mitosis (Figure 2b) was about 800 μm^2^. It is obvious that the actin stress fibers are more abundant on the larger spreading area, which may cause the larger elastic modulus.

## 4. Conclusions

In this paper, we quantitatively evaluated the elastic modulus of U-2 OS cells in the nuclear region and the peripheral region at the interphase and the telophase of mitosis, respectively. The results indicated that the stiffness of the peripheral region of the cell was significantly higher than that of other regions of the cell at either the interphase or the telophase of mitosis. However, the elastic modulus at the interphase was greater than that at the telophase of mitosis in the nuclear region and the peripheral region, respectively. This investigation reveals that the variation of the mechanical property can not only occur in a cancer cell, but also in a healthy cell which is at the division phase. Our findings in this study can contribute to providing more accurate cancer diagnoses by using the elastic modulus as a biomarker.
